# Economic impacts of the COVID-19 pandemic on families of children with autism and other developmental disabilities

**DOI:** 10.3389/fpsyt.2024.1342504

**Published:** 2024-02-14

**Authors:** Olivia M. Pokoski, Hayley Crain, Carolyn DiGuiseppi, Sarah M. Furnier, Eric J. Moody, Cy Nadler, Karen Pazol, Jessica Sanders, Lisa D. Wiggins, Maureen S. Durkin

**Affiliations:** ^1^ Department of Population Health Sciences, University of Wisconsin-Madison, Madison, WI, United States; ^2^ Waisman Center, University of Wisconsin-Madison, Madison, WI, United States; ^3^ Department of Pediatrics, University of Wisconsin-Madison, Madison, WI, United States; ^4^ Department of Epidemiology, Colorado School of Public Health, University of Colorado, Aurora, CO, United States; ^5^ Wyoming Institute for Disabilities, University of Wyoming, Laramie, WY, United States; ^6^ Division of Developmental and Behavioral Health, Children’s Mercy Kansas City, Kansas City, MO, United States; ^7^ National Center on Birth Defects and Developmental Disabilities, Centers for Disease Control and Prevention, Atlanta, GA, United States; ^8^ Department of Pediatrics and Department of Neurology, University of Colorado School of Medicine, Aurora, CO, United States

**Keywords:** COVID-19, autism spectrum disorder, neurodevelopmental disorder, COVID-19 pandemic impacts, family impacts

## Abstract

**Background:**

To control the spread of the coronavirus disease (COVID-19), many jurisdictions throughout the world enacted public health measures that had vast socio-economic implications. In emergency situations, families of children with developmental disabilities (DDs), including autism, may experience increased difficulty accessing therapies, economic hardship, and caregiver stress, with the potential to exacerbate autism symptoms. Yet, limited research exists on the economic impacts of the COVID-19 pandemic on families of children with autism or another DD compared to families of children from the general population.

**Objectives:**

To assess impact of the COVID-19 pandemic related to parental employment and economic difficulties in families of children with autism, another DD, and in the general population, considering potential modification by socioeconomic disadvantage before the pandemic and levels of child behavioral and emotional problems.

**Methods:**

The Study to Explore Early Development (SEED) is a multi-site, multi-phase, case-control study of young children with autism or another DD as compared to a population comparison group (POP). During January-July 2021, a COVID-19 Impact Assessment Questionnaire was sent to eligible participants (n=1,789) who had enrolled in SEED Phase 3 from September 2017-March 2020. Parents completed a questionnaire on impacts of the pandemic in 2020 and completed the Child Behavior Checklist (CBCL) to measure behavioral and emotional health of their child during this time. Multiple logistic regression models were built for employment reduction, increased remote work, difficulty paying bills, or fear of losing their home. Covariates include group status (autism, DD, POP), household income at enrollment, child’s race and ethnicity, and binary CBCL Total Problems T-score (<60 vs. ≥60). Unadjusted and adjusted odds ratios (aOR) and 95% confidence intervals (CI) were calculated.

**Results:**

The study included 274 children with autism, 368 children with another DD, and 385 POP children. The mean age of 6.1 years (standard deviation, 0.8) at the COVID-19 Impact Assessment did not differ between study groups. Parents of children with autism were less likely to transition to remote work (aOR [95% CI] = 0.6 [0.4, 1.0]) and more likely to report difficulty paying bills during the pandemic (1.8 [1.2, 2.9]) relative to parents of POP children. Lower income was associated with greater employment reduction, difficulty paying bills, and fear of losing their home, but inversely associated with transitioning to remote work. Parents of non-Hispanic (NH) Black children experienced greater employment reduction compared to parents of NH White children (1.9 [1.1, 3.0]). Parents from racial and ethnic minority groups were more likely to experience difficulty paying bills and fear losing their home, relative to NH White parents. Caregivers of children with CBCL scores in the clinical range were more likely to fear losing their home (2.1 [1.3, 3.4]).

**Conclusion:**

These findings suggest that families of children with autism, families of lower socio-economic status, and families of racial and ethnic minority groups experienced fewer work flexibilities and greater financial distress during the pandemic. Future research can be used to assess if these impacts are sustained over time.

## 1 Introduction

In March of 2020, a nationwide emergency was declared in the United States in an effort to control the spread of the coronavirus disease (COVID-19) (1). Many jurisdictions enacted stay-at-home orders, recommended quarantine protocols for healthy contacts of infected individuals, restricted personal travel, and temporarily closed non-essential businesses (2). These measures had vast socio-economic implications. Notably, the national unemployment rate increased to 13% from 3.8% at the beginning of the pandemic, with women losing employment at a significantly higher rate than men (3). The pandemic was also associated with increases in reported anxiety and depression and decreases in social participation and financial well-being (4).

Families of young children with autism and other developmental disabilities (DD) may have been more negatively affected by the pandemic restrictions than those of typically developing children. Autism is a neurodevelopmental disorder that is characterized by difficulties with social communication and social interaction as well as restricted and repetitive patterns in behaviors, interests, and activities (5). Children with autism may have a broad range of difficulties, including a strong preference for sameness, behavioral problems, and a need for assistance with activities of daily living. These difficulties may contribute to the higher levels of stress (6), depression and anxiety (7), and employment or financial insecurity (8, 9) reported by parents of children with autism. To mitigate these difficulties, many children with autism and other DDs see allied health professionals to help with addressing their various needs (10). Public health measures put in place to control the spread of COVID-19 disrupted the ability of some families to attend these appointments (11–13). Thus, the COVID-19 pandemic had the potential to exacerbate the potential stressors surrounding families of children with autism and other DDs.

Due to the recency of the COVID-19 pandemic, literature on its impact on families of children with autism or another DD is limited (14, 15). Some studies have documented intensified psychological and behavioral symptoms in children with autism (16–19), which could potentially be associated with increased parental stress or depression (17, 18, 20, 21). Several studies have found the COVID-19 pandemic to be associated with decreased quality of life or well-being for autistic individuals and their families (16, 22) and increased financial burden (21, 23), particularly in Black families (24). Other studies had conflicting results, with some families reporting perceived benefits of remote work or experiencing the pandemic as a welcome break, and others reporting that adjusting to the new normal with an autistic child exacerbated pre-existing challenges (25, 26). To facilitate effective planning for future public health emergencies, further research can help in understanding how families of children with autism as well as other DDs fared during the COVID-19 pandemic.

This study aimed to fill the aforementioned gaps in knowledge by surveying three groups of families of young children: those with a child with autism, those with a child with another DD, and those with a child sampled from the general population (POP). Our primary objectives were to identify differential impacts of the COVID-19 pandemic related to childcare, parental employment, and household financial security by group. We hypothesized that: (1) the impacts would be greater for families of children with autism or another DD than for POP children; (2) lower levels of socioeconomic status before the pandemic would exacerbate the adverse effects of the pandemic on families of children with disabilities; and (3) higher levels of child behavioral and emotional problems reported by parents would be associated with greater adverse impacts of the pandemic on families.

## 2 Methods

### 2.1 Study sample

Participants in this study originally completed the Study to Explore Early Development, Phase 3 (SEED3), a case-control study of young children funded by the Centers for Disease Control and Prevention (CDC) and implemented during 2017-2020 in six communities across the United States (Colorado, Georgia, Maryland, Missouri, North Carolina, and Wisconsin). SEED3 aimed to increase the understanding of autism and other DDs through the study of risk factors, co-occurring conditions, and behavioral phenotypes. Children were eligible for SEED3 if they were 2-5 years old, born and resided in the geographic study catchment area, and consistently lived with their biological mother from 6 months of age (or younger); additionally, the mother had to be able to communicate in English (or Spanish in the Colorado site). The mother participated in an extensive interview about sociodemographic characteristics, parental and child medical history, and services or treatments received. Additionally, several standardized assessments were performed by clinicians or completed by the mother.

Three groups of children were included: 1) children with autism; 2) children with another DD; and 3) POP children, as identified during their original participation in SEED3. Children were ascertained for potential inclusion in the autism or DD groups through sources serving or evaluating children with developmental difficulties, including but not limited to early intervention, special education, hospitals, and clinics. Participants who had previously received either an autism diagnosis from a clinical provider, or services for autism through early intervention or special education, or who scored ≥11 on the Social Communication Questionnaire (27) at enrollment, received a comprehensive developmental evaluation. A final autism classification was based on results from the Autism Diagnostic Observation Schedule (ADOS) (28, 29) and the Autism Diagnostic Interview-Revised (ADIR) (30, 31), administered in-person by research reliable clinicians. POP group children were identified through random sampling of birth records in the pre-specified birthdate geographic range at delivery. Prior publications document detailed descriptions of SEED eligibility criteria, enrollment methods, study group classification, and data collection (32–34).

The COVID-19 Impact Assessment was developed in 2020 to evaluate how changes related to the COVID-19 pandemic impacted children in SEED3 and their families. The 110-item questionnaire assessed changes in services and treatments, impacts on child development, changes in household routines, and impacts on household finances or parental employment during the pandemic in 2020. Families who completed SEED3 prior to March 31, 2020 were invited to participate in the COVID-19 Impact Assessment. Eligible families received the COVID-19 Impact survey along with a parent-report version of the Child Behavior Checklist (CBCL) (35) via mail and completed the items between January and June 2021. Parents who did not initially return the survey were contacted and provided with the option to complete the survey over the phone. The COVID-19 Impact Assessment was completed by the biological mother or father or another knowledgeable caregiver (hereinafter “parent” as 97.4% were completed by the mother or father).

The SEED3 protocol was approved by the CDC Institutional Review Board (IRB) and IRBs at each study site. In December 2020, the COVID-19 Impact Assessment was approved as an amendment to the SEED3 protocol. Reporting for this study was based on the Strengthening the Reporting of Observational Studies in Epidemiology guidelines (36).

### 2.2 Measures

All measures come from caregiver responses to the COVID-19 Impact Assessment. This assessment is published online (https://omb.report/icr/202102-0920-008/doc/108634700.pdf).

#### 2.2.1 Changes to childcare

Parents who reported having any type of childcare during January or February of 2020 were asked if changes related to COVID affected their regular childcare during 2020. This was a dichotomous variable in which respondents could report yes or no. Due to the skip pattern, those who did not have childcare pre-COVID did not answer this question and were not included in this outcome measure.

#### 2.2.2 Changes to parental employment

##### 2.2.2.1 Employment reduction

Parents who responded that they had a paying job in January or February of 2020 were asked if they lost their job permanently, temporarily, or had their work hours reduced due to changes related to COVID in 2020. If the respondent had a spouse or partner with a paying job during January or February of 2020, they were similarly asked if their spouse or partner lost their job permanently, temporarily, or had their work hours reduced due to changes related to COVID in 2020. Each question was reported as a dichotomous (yes or no) answer. Provided that either the respondent or their spouse or partner had a paying job in January or February of 2020, a ‘yes’ response to any of the questions above was coded as having employment reduced. If the parent responded ‘no’ to all the questions, they were coded as not having their employment reduced due to changes related to COVID. Parents who did not have a paying job in January or February 2020 and did not have a partner or did not have a partner who had a paying job in January or February 2020 were not included in this outcome measure.

##### 2.2.2.2 Increased remote work

Respondents with paying jobs in January or February 2020 were also asked if they either transitioned to remote work or increased hours worked remotely or from home due to COVID-related changes in 2020. Again, if the respondent had a spouse or partner with a paying job during January or February of 2020, they were asked the same questions. These four questions were consolidated into one variable, with a ‘yes’ response indicating that either the respondent or their partner either began working from home or increased hours worked from home. If all questions elicited ‘no’ responses, the respondent was coded as not working from home due to changes related to COVID. Respondents who did not have a paying job in January or February 2020 and did not have a partner or they had a partner who also did not have a paying job in January or February 2020 were not included in this outcome measure.

#### 2.2.3 Changes to household income

##### 2.2.3.1 Difficulty paying bills

Parents were asked how often they had difficulty paying their bills for each season of 2020 (i.e., pre-COVID: January-February; Spring: March-May; Summer: June-August; Fall: September-December). Response options were never, rarely, sometimes, very often, or extremely often. Never and rarely responses were combined, as were sometimes to extremely often due to small sample sizes. The seasons after the onset of the COVID-19 pandemic (Spring, Summer, and Fall) were also collapsed to gain an overall understanding of impacts during the pandemic. The response indicating the highest recorded difficulty throughout the seasons of the pandemic was used when consolidating variables.

##### 2.2.3.2 Fear of losing home

Parents were asked how often in each season of 2020 they feared they might lose their home due to a lack of money. Response options fell on a 5-point Likert scale ranging from never to extremely often. Binary coding for analytic purposes combined never with rarely responses versus sometimes to extremely often responses. Questions inquiring about fear during the Spring, Summer, and Fall were combined to form a variable indicating fear throughout the pandemic, with the response option indicating the highest frequency being reported for the variable.

#### 2.2.4 Child behavior checklist

The CBCL was completed by parents who received the invitation to participate in the COVID-19 Impact Assessment to determine the presence or absence of behavioral and emotional problems during the pandemic (35). Respondents were asked to characterize a list of child behaviors as “not true,” “somewhat or sometimes true,” or “very true or often true” for children ages 1.5-5. Parents completing the CBCL version for children 6-8 years characterized a list of child behaviors as “below average,” “average,” or “above average” compared to other children of the same age. Responses formed two broad scales: internalizing behavior problems and externalizing behavior problems, which made up an overall total problems scale. For this analysis, T-scores for total behavior problems (mean = 50, SD = 10) were converted to a binary variable. Scores of ≥60 indicate borderline to clinically significant behavior problems and scores <60 indicate no behavior problems compared to other children the same age.

### 2.3 Statistical analysis

Study sample characteristics were reported as numbers of observations (percentages) for categorical variables and means (standard deviations) for continuous variables. To assess differences between the autism, DD, and POP groups, likelihood ratio chi-square tests were performed for nominal descriptive variables, Mantel-Haenszel chi-square tests for ordinal variables, and one-way ANOVAs for continuous variables. Multivariable logistic regression was performed to assess associations between group status and impacts of the pandemic on parental employment (employment reduction and increased remote work) and household income related variables (difficulty paying bills and fear of losing home). Covariates for adjustment included group status (autism, DD, POP), family income at SEED3 relative to the federal poverty level (FPL) as a four-level categorical variable, child’s race and ethnicity, and binary CBCL Total Problems t-score. To evaluate potential effect modification by household income and behavioral problems, we performed logistic regression analyses stratified by the respective potential modifiers. We tested for an interaction between group status and household income relative to the FPL at SEED3 as well as between group status and binary CBCL Total Problems t-score. Bayesian information criteria (37) indicated that the models without interaction terms better fit the data and likelihood ratio tests were insignificant for most interaction terms (data not shown). Adjusted analyses were conducted using complete case analysis, removing 28 individuals (2.7%) with incomplete covariates.

Statistical analyses were performed using SAS software, Version 9.4 (38). All tests of statistical significance were two-tailed and a p-value of less than 0.05 was considered statistically significant.

## 3 Results

### 3.1 Sample characteristics

Of the 1,789 families from SEED3 invited to participate in the COVID-19 Impact assessment, 1,027 parents completed the survey (57.4%). Distribution of some key demographic characteristics stratified by study group are outlined in **Table 1**. At SEED3, children from the autism, DD, and POP groups significantly differed by demographic characteristics including, sex (p<0.0001), race and ethnicity (p=0.0018), and age (p<0.0001). Additionally, groups at SEED3 significantly differed by maternal education (p<0.0001), household income relative to the FPL (p<0.0001), health insurance status (p<0.0001) and study site (p=0.0082). There was not a statistically significant difference in the change in health insurance coverage between groups at the COVID-19 Impact Assessment (p=0.2955).

**Table 1:** Key characteristics of the Study to Explore Early Development COVID-19 Impact Assessment analytical sample.

	Autism (n = 274)	DD (n = 368)	POP (n = 385)	p-value^a^
Child sex, n (%)				<0.0001
Male	217 (79.2)	232 (63.0)	200 (52.0)	
Female	57 (20.8)	136 (37.0)	185 (48.1)	
Child race and ethnicity^b^, n (%)				0.0018
Hispanic	35 (12.8)	42 (11.4)	39 (10.1)	
NH, Black	41 (15.0)	43 (11.7)	24 (6.2)	
NH, Other or Multiracial	40 (14.6)	40 (10.9)	44 (11.4)	
NH, White	158 (57.7)	243 (66.0)	278 (72.2)	
Child age in years, mean (SD)				
At SEED3, from September 2017-March 2020	3.9 (0.7)	4.1 (0.8)	3.7 (0.7)	<0.0001
At COVID-19 Impact Assessment, from January-July 2021	6.1 (0.8)	6.2 (0.8)	6.1 (0.7)	0.1494
Maternal education at baseline, n (%)				<0.0001
< High school degree	<10	<10	<10	
High school degree	43 (15.7)	28 (7.6)	13 (3.4)	
Some college	81 (29.6)	63 (17.1)	61 (15.8)	
College graduate	89 (32.5)	118 (32.1)	136 (35.3)	
Advanced degree	52 (19.0)	150 (40.8)	171 (44.4)	
Missing	<10	<10	<10	
Household income relative to the FPL at SEED3, n (%)				<0.0001
≤138% FPL	72 (26.3)	58 (15.8)	28 (7.3)	
>138 to ≤250% FPL	58 (21.2)	56 (15.2)	76 (19.7)	
>250 to 400% FPL	84 (30.7)	112 (30.4)	113 (29.4)	
≥400% FPL	54 (19.7)	130 (35.3)	163 (42.3)	
Missing	<10	12 (3.3)	<10	
Health insurance status at SEED3, n (%)				<0.0001
Public insurance	100 (36.5)	76 (20.7)	57 (14.8)	
Private insurance	125 (45.6)	268 (72.8)	314 (81.6)	
Public & private insurance	47 (17.2)	23 (6.3)	<10	
No insurance	<10	<10	<10	
Health insurance changes at COVID-19 Impact Assessment, n (%)				0.2955
No change	260 (94.9)	351 (95.4)	355 (92.2)	
Increased coverage	<10	<10	10 (2.6)	
Decreased coverage	<10	11 (3.0)	20 (5.2)	
Study site, n (%)				0.0082
Colorado	39 (14.2)	52 (14.1)	79 (20.5)	
Georgi	31 (11.3)	59 (16.0)	35 (9.1)	
Maryland	39 (14.2)	35 (9.5)	40 (10.4)	
Missouri	59 (21.5)	61 (16.6)	82 (21.3)	
North Carolina	43 (15.7)	78 (21.2)	59 (15.3)	
Wisconsin	63 (23.0)	83 (22.6)	90 (23.4)	

n, sample size; DD, other developmental disabilities; POP, population comparison group; NH, non-Hispanic; SD, standard deviation.

a Likelihood ratio chi-square tests were used to calculate p-values for nominal descriptive variables, Mantel-Haenszel chi-square tests for differences in ordinal variables, and one-way ANOVA for continuous variables.

b Race and ethnicity data were collected by parent self-report and vital statistics/birth record data. Due to small sample sizes, American Indian/Alaska Native, Asian/Pacific Islander, or other race were combined into the NH other or multiracial race category.

### 3.2 Childcare changes

In January and February of 2020, 89.4% (autism group) to 92.9% (DD group) of the children in the study population attended a school or preschool outside of the home (**Table 2**). This percentage was not statistically different by group status (p=0.2903). However, not including preschool or school, only 28.2% of families of a child with autism reported having childcare in January or February 2020 before the onset of the COVID-19 pandemic, which was lower than families of children in the DD (42.7%) or POP (44.2%) groups. Despite a smaller percentage of families of children with autism reporting having childcare pre-COVID, a high percentage of participants in all groups responded that changes related to COVID affected their regular childcare (88.3%-91.8%; p=0.5395). Due to a small number of families with childcare pre-pandemic responding that their childcare was not affected by COVID, investigations by socioeconomic status or emotional and behavioral problems could not be made.

**Table 2:** Childcare, parental employment, and household income: Pre-COVID-19 and changes during the pandemic, Study to Explore Early Development COVID-19 Impact Assessment.

	Autism (n = 274)	DD (n = 368)	POP (n = 385)	p-value^a^
	n (%)	n (%)	n (%)	
Childcare changes				
Pre-COVID, child attended school/preschool	245 (89.4)	342 (92.9)	353 (91.7)	0.2903
Pre-COVID, family had other non-school childcare	77 (28.2)	157 (42.7)	170 (44.2)	<0.0001
Of those with childcare pre-COVID, changes related to COVID affected regular childcare	68 (88.3)	138 (88.5)	156 (91.8)	0.5395
Parental employment changes				
Paying job pre-COVID	255 (93.1)	355 (96.5)	377 (97.9)	0.0077
Employment reduction	118 (46.3)	129 (36.3)	156 (41.4)	0.0463
Increased remote work	149 (58.4)	248 (69.9)	294 (78.0)	<0.0001
Household Income Changes				
Difficulty paying bills, pre-COVID				<0.0001
Never or rarely	211 (77.0)	320 (87.0)	363 (94.3)	
Sometimes, very, or extremely often	63 (23.0)	48 (13.0)	22 (5.7)	
Difficulty paying bills during the pandemic in 2020				<0.0001
Never or rarely	175 (63.9)	278 (75.5)	331 (86.0)	
Sometimes, very, or extremely often	99 (36.1)	90 (24.5)	54 (14.0)	
Fear of losing home due to lack of money, pre-COVID				0.0019
Never or rarely	245 (89.7)	350 (95.1)	371 (96.4)	
Sometimes, very, or extremely often	28 (10.3)	18 (4.9)	14 (3.6)	
Fear of losing home during the pandemic in 2020				0.0002
Never or rarely	218 (79.9)	328 (89.1)	348 (90.4)	
Sometimes, very, or extremely often	55 (20.2)	40 (10.9)	37 (9.6)	

n, sample size; DD, other developmental disability; POP, population comparison group.

a Likelihood ratio chi-square tests were used to calculate p-values.

### 3.3 Parental employment changes

In the study sample, only 40 respondents reported neither they nor their partner had a paying job in January or February of 2020; 30 of these individuals reported not having a partner. There was a significant difference in the distribution of parents having a paying job by study group (**Table 2**, p=0.0077); families of children with autism had the lowest percentage, though for all three groups over 90% of respondents had a paying job pre-COVID. Nonetheless, participants in the autism group reported the greatest percentage of reduced work hours, permanently losing their job, or temporarily losing their job, due to changes related to COVID-19 in 2020 (46.3%). Parents also reported working remotely or from home at different percentages by group (p<0.0001), with parents of children with autism reporting the lowest percentage (58.4%), followed by parents of children with other DDs (69.9%), then parents of children in the POP group (78.0%).

However, in adjusted models, individuals in the DD group had significantly lower odds of reduced employment compared to the POP group (**Table 3**, aOR [95% CI] = 0.7 [0.5, 0.9]), but there was no significant difference in employment reduction between the autism and POP groups (0.8 [0.6, 1.2]). Families with lower household income experienced a significantly higher odds of having reduced employment due to the pandemic with decreasing household income categories, relative to those with a household income ≥400% of the FPL (**Table 3**). By race-ethnicity, parents of NH Black children had 90% higher odds of having reduced employment due to the pandemic, compared to NH White parents (1.9 [1.1, 3.0]). After adjustment for covariates, no difference was observed in employment reduction among families with children with and without behavioral problems as measured by the CBCL (1.2 [0.9, 1.7]).

**Table 3:** Odds ratios for parental employment changes during 2020 of the COVID-19 pandemic, Study to Explore Early Development COVID-19 Impact Assessment.

Variable	Respondent or partner had a paying job, pre-COVID	Employment reduction: respondent or partner reduced work hours, or lost job permanently or temporarily			Increased remote work: respondent or partner started working remotely or increased hours working remotely		
	N (% yes)	N (% yes)	Unadjusted OR (95% CI)	Adjusted OR^a^ (95% CI)	N (% yes)	Unadjusted OR (95% CI)	Adjusted OR^a^ (95% CI)
Group status							
Autism	255 (93.1)	118 (46.3)	1.2 (0.9, 1.7)	0.8 (0.6, 1.2)	149 (58.4)	**0.4 (0.3, 0.6)**	**0.6 (0.4, 1.0)**
DD	355 (96.5)	129 (36.3)	0.8 (0.6, 1.1)	**0.7 (0.5, 0.9)**	248 (69.9)	**0.7 (0.5, 0.9)**	0.8 (0.5, 1.2)
POP	377 (97.9)	156 (41.4)	1.0	1.0	294 (78.0)	1.0	1.0
Income							
≤138% FPL	128 (81.0)	77 (60.2)	**3.3 (2.2, 5.1)**	**3.0 (1.9, 4.8)**	28 (21.9)	**<0.1 (0.0, 0.1)**	**<0.1 (0.0, 0.1)**
>138 to ≤250% FPL	186 (97.9)	88 (47.3)	**2.0 (1.4, 2.9)**	**1.9 (1.3, 2.7)**	105 (56.5)	**0.2 (0.1, 0.3)**	**0.2 (0.1, 0.3)**
>250 to 400% FPL	308 (99.7)	120 (39.0)	**1.4 (1.0, 1.9)**	**1.4 (1.0, 2.0)**	245 (79.6)	**0.6 (0.4, 0.9)**	**0.6 (0.4, 0.9)**
≥400% FPL	343 (98.9)	107 (31.2)	1.0	1.0	300 (87.5)	1.0	1.0
Race and ethnicity^b^							
Hispanic	111 (95.7)	45 (40.5)	1.1 (0.8, 1.7)	0.9 (0.6, 1.3)	67 (60.4)	**0.5 (0.3, 0.8)**	1.0 (0.6, 1.6)
NH, Black	94 (87.0)	58 (61.7)	**2.7 (1.7, 4.2)**	**1.9 (1.1, 3.0)**	45 (47.9)	**0.3 (0.2, 0.5)**	0.6 (0.4, 1.1)
NH, Other or Multiracial	116 (93.6)	50 (43.1)	1.3 (0.8, 1.9)	1.2 (0.8, 1.8)	78 (67.2)	0.7 (0.4, 1.0)	0.7 (0.4, 1.1)
NH, White	666 (98.1)	250 (37.5)	1.0	1.0	501 (75.2)	1.0	1.0
CBCL Total Score							
≥60 Clinical range	272 (92.2)	129 (47.4)	**1.5 (1.1, 1.9)**	1.2 (0.9, 1.7)	164 (60.3)	**0.5 (0.4, 0.7)**	1.1 (0.8, 1.7)
<60 Normal range	711 (97.7)	271 (38.1)	1.0	1.0	524 (73.7)	1.0	1.0

OR, odds ratio; CI, confidence interval; ASD, autism spectrum disorder; DD, other developmental disability; POP, population control participants; FPL, Federal Poverty Line; NH, Non-Hispanic; CBCL, Child Behavior Checklist.

a Odds ratios presented by each variable (group status, 4-category income relative to the federal poverty level, race and ethnicity, and binary CBCL total score) are adjusted for the remaining variables listed in the table.

b Due to small sample sizes, Asian or Pacific Islander/Native Hawaiian, American Indian/Alaskan Native, those reporting another race, or of multiple races were combined to form the NH Other or Multiracial category.

Bold indicates statistical significance at alpha = 0.05.

Respondents in the autism and DD groups who reported having a paying job or a partner with a paying job in January or February of 2020, had lower odds of beginning to work remotely or from home or increasing hours worked remotely or from home (autism group: 0.4 [0.3, 0.6], DD group: 0.7 [0.5, 0.9]) relative to the POP group. These results were attenuated after adjusting for covariates (0.6 [0.4, 1.0] for the autism group and 0.8 [0.5, 1.2] for the DD group). Each income category below 400% of the FPL had significantly lower odds of increasing remote work hours with the COVID-19 pandemic (**Table 3**). After adjusting for covariates, no differences were observed between increased remote work in minority families relative to NH White families, or between families of children with or without clinically significant CBCL scores.

### 3.4 Household income changes

Pre-COVID and during the COVID-19 pandemic, higher percentages of respondents with a child in the autism and DD groups reported having difficulty paying bills sometimes, very, or extremely often compared to the POP group (**Table 2**). **Table 4** depicts the unadjusted and covariate-adjusted results from the multivariable logistic regression models for difficulty paying bills during the COVID-19 pandemic in 2020. After adjusting for covariates, families of children in the autism group had 80% higher odds (1.8 [1.2, 2.9]) of reporting difficulty paying bills during the pandemic compared to the POP group. Similarly, families of children with other DDs had 60% higher odds of reporting difficulty paying bills during the pandemic compared to the POP group after adjustment (1.6 [1.0, 2.5]). By income, those with the lowest income relative to the FPL at SEED3 had the highest odds of difficulty paying bills (12.6 [7.3, 22.0]). Furthermore, each household income category relative to the FPL had significantly higher odds of difficulty paying bills during the pandemic compared to those with income ≥400% FPL, with decreasing magnitude as income increased (**Table 4**). Parents from racial and ethnic minority groups were more likely to experience difficulty paying bills during the pandemic, with parents of NH Black children experiencing the most difficulty compared to NH White children (3.6 [2.2, 6.0]). After adjustment for covariates, difficulty paying bills during the pandemic did not differ between families of a child with a clinically significant CBCL score relative to those with a child with a CBCL score <60 (1.3 [0.9, 1.9]).

**Table 4:** Odds ratios for household income changes during 2020 of the COVID-19 pandemic, Study to Explore Early Development COVID-19 Impact Assessment.

	Difficulty paying bills				Fear of losing home			
Variable	Pre-COVID N (%)	Pandemic N (%)	Unadjusted OR (95% CI)	Adjusted OR^a^ (95% CI)	Pre-COVID N (%)	Pandemic N (%)	Unadjusted OR (95% CI)	Adjusted OR^a^ (95% CI)
Group Status								
Autism	63 (23.0)	99 (36.1)	**3.5 (2.4, 5.1)**	**1.8 (1.2, 2.9)**	28 (10.3)	55 (20.2)	**2.4 (1.5, 3.7)**	0.9 (0.5, 1.6)
DD	48 (13.0)	90 (24.5)	**2.0 (1.4, 2.9)**	**1.6 (1.0, 2.5)**	18 (4.9)	40 (10.9)	1.1 (0.7, 1.8)	0.7 (0.4, 1.3)
POP	22 (5.7)	54 (14.0)	1.0	1.0	14 (3.6)	37 (9.6)	1.0	1.0
Income								
≤138% FPL	62 (39.2)	95 (60.1)	**20.3 (12.0, 34.2)**	**12.6 (7.3, 22.0)**	34 (21.7)	56 (35.7)	**18.7 (9.2, 38.0)**	**11.0 (5.2, 23.2)**
>138 to ≤ 250% FPL	34 (17.9)	63 (33.2)	**6.7 (4.0, 11.2)**	**5.9 (3.5, 10.1)**	11 (5.8)	29 (15.3)	**6.1 (2.9, 12.8)**	**5.0 (2.4, 10.8**)
>250 to 400% FPL	24 (7.8)	50 (16.2)	**2.6 (1.6, 4.3)**	**2.5 (1.5, 4.2)**	<10	30 (9.7)	**3.6 (1.7, 7.5)**	**3.5 (1.6, 7.3)**
≥400% FPL	<10	24 (6.9)	1.0	1.0	<10	10 (2.9)	1.0	1.0
Race and Ethnicity^b^								
Hispanic	22 (19.0)	39 (33.6)	**2.6 (1.7, 4.1)**	1.5 (0.9, 2.5)	10 (8.6)	23 (19.8)	**3.2 (1.8, 5.5)**	**2.0 (1.1, 3.6)**
NH, Black	41 (38.0)	63 (58.3)	**7.2 (4.7, 11.2)**	**3.6 (2.2, 6.0)**	27 (25.0)	38 (35.2)	**7.0 (4.3, 11.4)**	**3.8 (2.2, 6.7)**
NH, Otheror Multiracial	12 (9.7)	31 (25.0)	**1.7 (1.1, 2.7)**	1.5 (0.9, 2.5)	<10	22 (17.7)	**2.8 (1.6, 4.8)**	**2.6 (1.4, 4.7)**
NH, White	58 (8.5)	110 (16.2)	1.0	1.0	16 (2.4)	49 (7.2)	1.0	1.0
CBCL Total Score								
≥60Clinical range	70 (23.7)	107 (36.3)	**2.5 (1.9, 3.4)**	1.3 (0.9, 1.9)	39 (13.3)	69 (23.5)	**3.3 (2.3, 4.8)**	**2.1 (1.3, 3.4)**
<60Normal range	62 (8.5)	134 (18.4)	1.0	1.0	20 (2.8)	62 (8.5)	1.0	1.0

OR, odds ratio; CI, confidence interval; ASD, autism spectrum disorder; DD, other developmental disability; POP, population control participants; FPL, Federal Poverty Line; NH, Non-Hispanic; CBCL, Child Behavior Checklist.

a Odds ratios presented by each variable (group status, 4-category income relative to the federal poverty level, race and ethnicity, and binary CBCL total score) are adjusted for the remaining variables listed in the table.

b Due to small sample sizes, Asian or Pacific Islander/Native Hawaiian, American Indian/Alaskan Native, those reporting another race, or those reporting more than one race were combined to form the non-Hispanic Other or Multiracial category.

Bold indicates statistical significance at alpha = 0.05.

The percentages of respondents who reported that they feared losing their home sometimes, very, or extremely often during the COVID-19 pandemic differed across groups, with the highest percentage in the autism group (20.2%) and the lowest percentage in the POP group (9.6%, **Table 2**). After adjusting for covariates, there was no significant difference in fear of losing one’s home during the COVID-19 pandemic between the autism (0.9 [0.5, 1.6]) or DD (0.7 [0.4, 1.3]) groups relative to the POP group. Relative to families with a household income ≥400% of the FPL, families of lower income had higher odds of being fearful of losing their home during the pandemic, with increasing magnitude as household income decreased (**Table 4**). Families from racial and ethnic minority groups were more fearful of losing their homes during the pandemic compared to families of NH White children, after adjusting for covariates (**Table 4**). Caregivers of children who have clinically significant behavioral and emotional health problems had 2.1 times higher odds (95% CI: 1.3, 3.4) of being fearful of losing their home during the pandemic compared to those with children with CBCL score <60.

### 3.5 Potential effect modifiers

The odds ratios indicating impacts of the pandemic on families of children with autism relative to families in the POP group were similar for lower income (≤200% FPL) and higher income (>200% FPL) families (**Table 5**). In contrast, the odds ratios comparing the DD group to the POP group differed somewhat by income level. In families of higher income, the DD group was less likely to experience employment reduction compared to the POP group (0.6 [0.4, 0.9]), whereas in lower income families, parents of children with other DDs had similar employment reduction compared to the POP group (1.1 [0.5, 2.3]). Additionally, in lower income families, those in the DD group had an increased odds of difficulty paying bills (2.2 [1.1, 4.4]) compared to families of children in the POP group, whereas, among higher income families, the odds of having difficulty paying bills was similar in the DD and POP groups (1.3 [0.8, 2.2]). The likelihood ratio test for the group status by household income interaction was only statistically significant for the logistic regression model examining parents who increased remote work during the pandemic (p=0.0422). However, model coefficients and resulting odds ratios were similar with or without the inclusion of this interaction term (data not shown).

**Table 5:** Odds ratios relative to the POP group for parental employment changes and household income changes during 2020 of the COVID-19 pandemic stratified by binary household income at SEED3, Study to Explore Early Development COVID-19 Impact Assessment .

	Autism		DD	
	≤200% FPL aOR^a^ (95% CI)	>200% FPL aOR (95% CI)	≤200% FPL aOR (95% CI)	>200% FPL aOR (95% CI)
Employment reduction	0.8 (0.4, 1.6)	1.0 (0.7, 1.6)	1.1 (0.5, 2.3)	**0.6 (0.4, 0.9)**
Increased remote work	**0.5 (0.2, 1.0)**	0.6 (0.4, 1.1)	0.6 (0.3, 1.2)	0.9 (0.6, 1.4)
Difficulty paying bills	**2.2 (1.1, 4.5)**	**1.9 (1.0, 3.4)**	**2.2 (1.1, 4.4)**	1.3 (0.8, 2.2)
Fear of losing home	1.2 (0.5, 2.7)	0.9 (0.4, 2.0)	1.0 (0.4, 2.2)	0.6 (0.3, 1.3)

POP, child sampled from the general population; SEED3, Study to Explore Early Development, Phase 3; DD, developmental disability; FPL, federal poverty level;

aOR, adjusted odds ratio; CI, confidence interval, NH, non-Hispanic.

a Logistic regression models include the following covariates: group status (autism, DD, POP), child’s race and ethnicity (NH Black, Hispanic, NH Other or Multiracial, NH White), and binary Child Behavior Checklist Total Problems t-score (≥60 or <60).

Bold indicates statistical significance at alpha <0.05.

We found no evidence of effect modification in analyses stratified by CBCL Total Problems t-score categories (**Table 6**). In families of children with autism and families of children with other DDs compared to the POP group, the odds ratios were largely similar for families of children with typical CBCL scores (<60) and those scoring in the borderline to clinically significant range (≥60). For each outcome, likelihood ratio tests for the interaction between binary CBCL Total Problems t-score and group status were not statistically significant (data not shown).

**Table 6:** Odds ratios relative to the POP group for parental employment changes and household income changes during 2020 of the COVID-19 pandemic stratified by CBCL Total Problems binary score, Study to Explore Early Development COVID-19 Impact Assessment.

	Autism		DD	
	CBCL <60 aOR^a^ (95% CI)	CBCL ≥60 aOR (95% CI)	CBCL <60 aOR (95% CI)	CBCL ≥60 aOR (95% CI)
Employment reduction	0.8 (0.5, 1.3)	0.7 (0.3, 1.6)	0.7 (0.5, 1.0)	0.5 (0.2, 1.2)
Increased remote work	0.6 (0.4, 1.0)	0.6 (0.2, 1.6)	0.9 (0.6, 1.3)	0.7 (0.2, 1.8)
Difficulty paying bills	**2.0 (1.1, 3.5)**	1.8 (0.7, 4.6)	1.5 (0.9, 2.5)	1.8 (0.7, 5.0)
Fear of losing home	1.0 (0.5, 2.1)	0.6 (0.2, 1.7)	0.9 (0.5, 1.8)	0.4 (0.1, 1.2)

POP, child sampled from the general population; CBCL, Child Behavior Checklist; DD, developmental disability; aOR, adjusted odds ratio; CI, confidence interval, NH, non-Hispanic.

a Logistic regression models include the following covariates: group status (autism, DD, POP), household income relative to the federal poverty level (FPL) at SEED3 (≤138% FPL, > 138 to ≤250% FPL, >250 to 400% FPL, ≥400% FPL), and child’s race and ethnicity (NH Black, Hispanic, NH Other or Multiracial, NH White).

Bold indicates statistical significance at alpha <0.05.

## 4 Discussion

Our study examined impacts of the COVID-19 pandemic on measures of childcare, parental employment, and household income on a sample of families of three groups of children: those with autism, those with another DD, and a population comparison group. Even after adjusting for household income, race and ethnicity, and the behavioral and emotional problems of the child, families of children with autism reported increased odds of difficulty paying bills and decreased odds of working remotely or from home compared to families of POP children. Targeted support systems may need to be established in anticipation of future emergency situations to assist vulnerable populations, including families of children with disabilities.

We found parents of children in the autism group were less likely to have a paying job and had greater difficulty paying bills prior to the onset of the pandemic, when compared to parents of children in the POP group. This finding is consistent with previous studies documenting the economic impacts of autism, including decreased parental employment and productivity due to therapy schedules and caregiving needs as well as the high out-of-pocket costs of autism services and the excess healthcare expenditures in general for children with autism relative to children without disabilities (39–45). However, our overall sample had a high frequency of parents with a paying job pre-COVID, as 93% of parents of children with autism reported having a paying job. Still, we attempted to mitigate potential confounding by adjusting for household income at SEED3. While some previous studies reported that parents of children with either autism or another DD had financial concerns due to the pandemic, these studies lacked a comparison group, making it difficult to determine if financial concerns were universal or associated with having a child with a DD (21, 23). Simply examining frequencies, each group had an elevated difficulty paying bills during the pandemic, which aligns with the findings of these studies.

We found that families of children with autism had 40% lower odds of working remotely or from home during the COVID-19 pandemic after adjusting for race and ethnicity, income, and behavioral and emotional problems, compared to families of POP children. Previous literature cites remote work and parenting a child with autism as increasing parental stress due to the difficulty of balancing work obligations and facilitating the child’s therapies and schoolwork (23, 25), and parents who switched to working from home felt that the pandemic increased their responsibilities (46). This perceived increase in stress and responsibility may help explain why parents of children with autism were less likely to work from home during the pandemic in this study, though the data do not allow us to determine the reason for being less likely to work remotely. The increased demands on working parents with the COVID-19 pandemic were not specific to parents of children with autism, as research indicates that many parents of children without disabilities also experienced difficulties balancing remote work and caregiving (47, 48). In developing preparations for future public health emergencies, it may be important to recognize that parents of children with autism may face additional challenges in working from home.

An abundance of research has documented how the pandemic disproportionately impacted lower income families (49–52). After adjusting for group status, race and ethnicity, and behavioral and emotional problems, families with a lower household income prior to the pandemic had significantly greater odds of employment reduction, difficulty paying bills, and fear of losing their home during the pandemic relative to families in the highest income category. There are likely additional factors that contributed to the disproportionate disadvantages faced by families with lower income that we could not measure. Individuals with a lower household income pre-pandemic also had lower odds of working remotely or from home than those with a higher household income. While it is possible that individuals of lower income were working jobs that could not be performed remotely, a lack of resources such as a computer or internet accessibility might have also served as a barrier.

We hypothesized lower levels of socioeconomic status before the pandemic would exacerbate the adverse effects of the pandemic on families of children with disabilities. However, we found little evidence that the impact of having a child with autism or other disabilities on COVID-related measures of parental employment and household income changes was greater for low income than high income families.

Several studies have highlighted the impacts of the pandemic on behavioral problems in children with autism (16–18, 53, 54). In light of this, we hypothesized that higher levels of child behavioral and emotional problems would be associated with greater adverse economic and employment impacts on their families during the pandemic. We found that families of children with behavioral problems in the clinical range were more than twice as likely to report fear of losing their home compared to families of children without behavioral problems. Prior to the pandemic, in non-emergency settings, research indicated that parental stress is a consequence and antecedent of child behavior problems, while simultaneously, child behavior problems are both an antecedent and consequence of parental stress (55). Evidence supporting this idea during the COVID-19 pandemic has been documented as the “spillover hypothesis” that suggests that high levels of stress or anxiety in parents can “spill over” to their children and lead to an increase in child behavior problems (56–58). It is possible that the increased economic stressors of the pandemic may have exacerbated the emotional and behavioral problems in children. It is also possible that parents who reported being fearful of losing their home had increased stress relative to those who were not fearful of losing their home. However, we did not find evidence that the economic impact of having a child with autism or other DD differed by the presence or absence of behavior problems in the child.

We found that families of racial and ethnic minority groups experienced greater adverse economic impacts than other families. These findings align with previous research suggesting that Black and Hispanic individuals had more frequent job or wage loss and more trouble paying bills compared to White individuals during the pandemic (59–61). The disproportionate socioeconomic impact of the COVID-19 pandemic on racial and ethnic minority groups may have only worsened the disparities that are already present (62). Initiatives to assist communities that may experience disproportionate impacts may help mitigate the heightened negative impacts in future public health emergencies.

### 4.1 Limitations

At SEED3, the groups were not balanced on key variables of interest, with families of children in the autism and DD groups being of a lower socioeconomic status than families of POP children. We attempted to eliminate the potential confounding by socioeconomic status by adjusting the models for household income at SEED3, but residual confounding is possible. Additionally, by conducting a complete case analysis, individuals who had missing data in any of the outcomes or covariates were excluded from the analysis. However, only 2.5% of the sample had a missing variable so it is likely that the results would remain unchanged if these individuals were to be included. Finally, our analyses relied on self-reported measures of employment and income changes during 2020 from the COVID-19 Impact Assessment which was completed between January and June of 2021, which may have introduced measurement bias in the form of social desirability, recall bias, or errors in reporting.

### 4.2 Conclusion

Families of children with autism and other DDs may be especially vulnerable in public health emergencies. Using data from the COVID-19 Impact Assessment, we found that families of children with autism more often reported difficulty paying bills and lower odds of transitioning to remote work during 2020 of the pandemic compared to families of children sampled from the general population. We also found that families of lower socio-economic status as well as families of racial and ethnic minority groups experienced fewer work flexibilities and greater financial distress during the pandemic. Future research can help us better understand if these impacts are sustained over time and how best to support families who might lie at the intersection of these disadvantages during future emergency situations.

## Data availability statement

The original data presented in this paper are protected to ensure participant confidentiality. Further inquiries can be directed to the corresponding author.

## Ethics statement

The studies involving humans were approved by The SEED3 protocol, CDC Institutional Review Board (IRB) and IRBs at each study site. In December 2020, the SEED3 COVID-19 Impact Assessment was approved as an amendment to the SEED3 protocol. The studies were conducted in accordance with the local legislation and institutional requirements. Written informed consent for participation in this study was provided by the participants’ legal guardians/next of kin.

## Author contributions

OP: Conceptualization, Data curation, Formal analysis, Investigation, Methodology, Writing – original draft, Writing – review & editing. HC: Methodology, Supervision, Writing – review & editing. CD: Funding acquisition, Investigation, Supervision, Writing – review & editing. SF: Data curation, Investigation, Methodology, Validation, Writing – review & editing. EM: Writing – review & editing. CN: Investigation, Supervision, Writing – review & editing. KP: Funding acquisition, Investigation, Supervision, Writing – review & editing. JS: Writing – review & editing. LW: Investigation, Methodology, Supervision, Writing – review & editing. MD: Conceptualization, Data curation, Formal analysis, Funding acquisition, Investigation, Methodology, Project administration, Resources, Supervision, Writing – review & editing.
